# Covid-19 y comunidades diaguitas: desigualdades estructurales y estrategias (inter)comunitarias en el departamento Santa María (Catamarca, Argentina)

**DOI:** 10.18294/sc.2024.4868

**Published:** 2024-10-18

**Authors:** Luciana García Guerreiro

**Affiliations:** 1 Doctora en Ciencias Sociales, Becaria posdoctoral CONICET, Grupo de Estudios Rurales, Grupo de Estudios de los Movimientos Sociales de América Latina (GER-GEMSAL), Instituto de Investigaciones Gino Germani (IIGG), Universidad de Buenos Aires, Argentina. lucianagarciaguerreiro@yahoo.com.ar Universidad de Buenos Aires Grupo de Estudios de los Movimientos Sociales de América Latina (GER-GEMSAL) Instituto de Investigaciones Gino Germani (IIGG) Universidad de Buenos Aires Argentina lucianagarciaguerreiro@yahoo.com.ar

**Keywords:** Salud de los Pueblos Indígenas, Salud Pública, COVID-19, Territorio Sociocultural, Medicina Tradicional, Argentina

## Abstract

El objetivo fue indagar y analizar el impacto social, sanitario y económico de la pandemia covid-19 en comunidades indígenas de la provincia de Catamarca (Argentina), buscando identificar también las diferentes estrategias construidas por parte de estas comunidades para hacer frente a este fenómeno. El trabajo de investigación se llevó a cabo entre diciembre de 2021 y diciembre de 2022, y ha tenido un carácter exploratorio y un diseño metodológico predominantemente cualitativo. Durante el trabajo de campo, se llevaron a cabo 15 entrevistas en profundidad a autoridades comunitarias, personas de la comunidad, técnicos territoriales y agentes de salud, las cuales fueron complementadas con 30 entrevistas estructuradas realizadas a personas de diferentes comunidades del departamento Santa María. Si bien se evidencia la existencia de tensiones entre el sistema de salud oficial y las comunidades indígenas (con sus cosmovisiones y sus prácticas ancestrales), también se observa la relevancia que ha asumido la autogestión comunitaria y el control territorial de los procesos organizativos indígenas en las acciones de prevención y atención de la salud, y en el establecimiento de diálogos y negociaciones con las autoridades del sistema de salud local respecto a la implementación de medidas en los territorios.

## INTRODUCCIÓN

El surgimiento del covid-19 y su expansión pandémica ha repercutido profundamente en todos los aspectos que hacen a la reproducción social. La implementación de medidas de aislamiento social preventivo que fueron adoptadas por algunos gobiernos -como en el caso de Argentina-, necesarias para prevenir y mitigar el avance de los contagios, tuvieron efectos en la vida social, profundizando las insondables consecuencias, tanto en aspectos económicos, sociales, psicológicos, como ambientales que la pandemia ha representado para la población.

Los pueblos indígenas, por encontrarse en situación de desigualdad estructural, tanto socioeconómica como sanitaria y cultural, han sufrido en mayor grado los efectos de la pandemia de covid-19 y las medidas de confinamiento social^1^^,^^2^^,^^3^. Al respecto, resulta importante comprender a los pueblos indígenas no como sectores vulnerables *per se*, sino como sujetos individuales y colectivos cuyos derechos han sido sistemáticamente vulnerados^4^ en el marco de matrices coloniales de poder y saber^5^^,^^6^^,^^7^. De ese modo, la vulnerabilidad puede comprenderse como resultante de procesos históricos y conflictos sociales, que “se traducen en accesibilidades diferenciales a los recursos materiales y simbólicos necesarios para poder practicar el autocuidado y el cuidado, para la promoción de la salud, para la prevención de los contagios, las enfermedades y las muertes”^4^.

La presente investigación se propuso problematizar el modo en que la pandemia de covid-19 ha impactado en comunidades diaguitas en el departamento Santa María (provincia de Catamarca, Argentina), pero también dar cuenta de las diferentes estrategias construidas por parte de estas comunidades para hacer frente al fenómeno, en el marco de luchas territoriales por el reconocimiento de derechos. En este punto, se ponderan los aspectos comunitarios y organizativos indígenas que permiten a los pueblos enfrentar la presencia de nuevas situaciones adversas, que se suman a los históricos procesos de expropiación, arrinconamiento y exterminio que padecen. En tal sentido, consideramos que las experiencias de lucha, las formas organizativas y la dimensión comunitaria que hacen a la resistencia y re-existencia^8^ de los pueblos indígenas en la región constituyen un elemento ineludible para el análisis acerca de los efectos de la pandemia de covid-19 y de las medidas adoptadas. Al respecto, cabe destacar que, durante las últimas décadas, en el marco de procesos de “emergencia indígena”^9^, las comunidades diaguitas bajo estudio han profundizado su lucha por el territorio, la reorganización política de sus comunidades y el reconocimiento de sus derechos ancestrales^10^.

En este punto, consideramos central incorporar la noción de territorio, entendiendo al territorio como un espacio geográfico atravesado por relaciones sociales, políticas, culturales y económicas que es resignificado constantemente por los actores que lo habitan y hacen uso de él, configurando un escenario territorial en conflicto por la apropiación y reterritorialización del espacio y los bienes naturales, propiciando la formación de identidades (territorialidades)^11^. En ese sentido, dichas prácticas y estrategias que llevan a cabo las comunidades indígenas se inscriben como parte de procesos de apropiación y territorialización del espacio de vida y, por lo tanto, asumen una importancia central en el marco de los procesos de defensa y reconocimiento étnico territorial que actualmente atraviesan. Así, la relación entre salud y territorio resulta fundamental.

Por esa misma razón, comprendemos que la salud está integrada a todos los fenómenos de la vida y que, por eso mismo, como señala Cuyul Soto^12^, atiende a condicionamientos económicos, políticos, ambientales, espirituales, culturales e históricos. Muchas acciones de los programas de salud para pueblos indígenas se han concentrado en atender necesidades puntuales -que precisamente son producto de las brechas estructurales y sociohistóricas- desde enfoques paternalistas y/o asistencialistas, que lejos están de favorecer el ejercicio de autonomía y el derecho a la autodeterminación de los pueblos indígenas^12^^,^^13^^).^

Respecto a los efectos de la pandemia por covid-19, en comunidades indígenas encontramos que, si bien se han presentado diferentes informes que abordan la problemática a nivel nacional^1^^,^^4^^,^^14^^,^^15^, no existe información oficial sobre los casos de covid-19 entre los pueblos indígenas de nuestro país, exponiendo las dificultades para aplicar un enfoque étnico en los sistemas de información sanitarias^2^. Ante la carencia de este tipo de abordajes e información específica en la provincia de Catamarca, nos hemos propuesto tomar como caso seis comunidades diaguitas del departamento Santa María (Catamarca), preguntándonos no solo por el modo en que la pandemia por covid-19 ha impactado en términos sanitarios, sociales y económicos en comunidades rurales, e indígenas en particular, sino también por las diferentes estrategias construidas por parte de estas comunidades para hacer frente a este fenómeno. 

En ese marco, algunas preguntas que orientaron el trabajo han sido: ¿Cómo han afectado las situaciones de desigualdad estructural e histórica^13^ en el impacto de la pandemia covid-19?; ¿cuál ha sido el impacto de las medidas sanitarias de aislamiento social preventivo y obligatorio (ASPO) y de otras políticas públicas implementadas a raíz del contexto de pandemia y cómo han sido recibidas por las comunidades?; ¿qué políticas de salud se han implementado en los territorios diaguitas catamarqueños para atender las necesidades sanitarias de dichas comunidades?; ¿qué percepciones y estrategias comunitarias se han construido desde los territorios indígenas frente a este fenómeno?

### Descripción del contexto de investigación

La investigación ha centrado su atención en las comunidades diaguitas del departamento Santa María (Catamarca), en particular, las comunidades: Cerro Pintao (40 familias), Famabalasto (30 familias), La Quebrada (12 familias), La Hoyada (40 familias), Toro Yaco (18 familias) y Alto Valle El Cajón (79 familias). Según el último Censo Nacional de Población, Hogares y Viviendas, en la provincia de Catamarca, en 2022, de un total de 427.625 habitantes, 19.668 se reconocían indígenas o descendientes de indígenas, lo que representa el 4,6% de la población. Por su parte, en Santa María, 5.465 personas se reconocían indígenas de un total de 26.822 habitantes en el departamento. Es decir, en 2022, el 20,4% de la población del departamento se reconocía indígena. Si tenemos en cuenta la dimensión organizativa, existen en la provincia más de veinte comunidades diaguitas, de las cuales nueve se encuentran en el departamento Santa María. De esas nueve, a los fines de la investigación, se ha trabajo con seis comunidades, las cuales, a su vez, se encuentran agrupadas a nivel provincial y regional en una organización de pueblos indígenas de segundo grado (Unión de los Pueblos de la Nación Diaguita de Catamarca). Este es un elemento que consideramos significativo en términos organizativos y territoriales.

**Figura 1:**
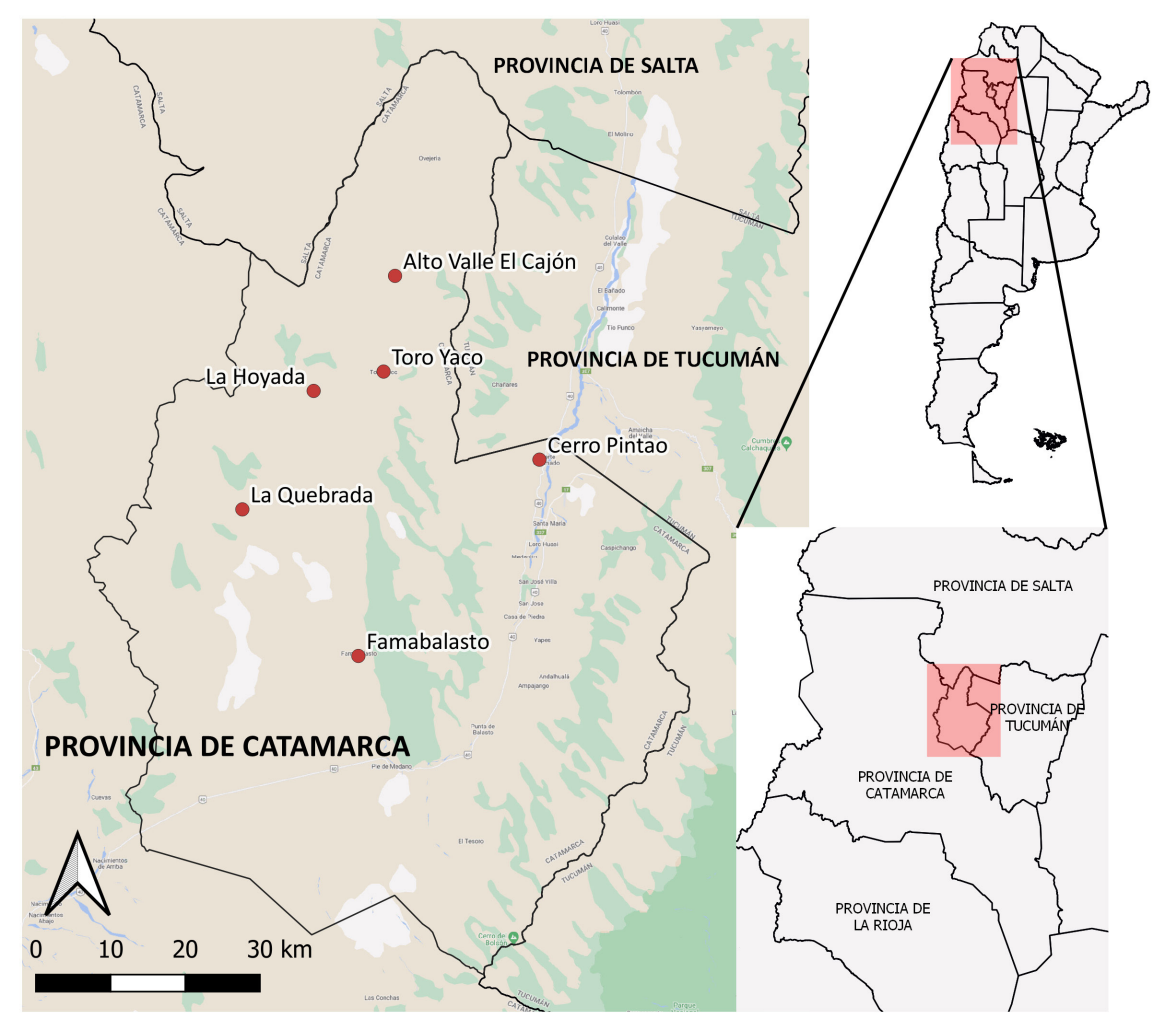
Ubicación de las comunidades diaguitas bajo estudio. Departamento Santa María, Catamarca, Argentina, 2022.

Las comunidades se encuentran ubicadas mayormente en lo que se denomina zona serrana, es decir, los cerros y zonas altas del Valle de Yokavil y el Alto Valle del Cajón a una altura de entre 1.600 y 4.500 msnm (Figura 1), aunque gran parte de las familias comuneras mantienen un vínculo constante y frecuente con la zona más baja, que se encuentra más urbanizada y en la que se concentra una buena parte de las actividades político-administrativas, comerciales, establecimientos educativos y centros de salud del departamento. 

Fuente: Elaboración propia.

Al momento de la investigación, según información brindada por las comunidades, eran 219 las familias censadas en las seis comunidades bajo estudio, lo que representa, en total, cerca de 700 personas aproximadamente. Cabe mencionar que la investigación se enmarca en vínculos construidos previamente con las comunidades, ya sea en trabajos de investigación anteriores^10^^,^^13^ como en acciones que exceden los objetivos de la investigación (por realizar acompañamientos técnicos socio-organizativos), lo cual constituye un elemento fundamental para el trabajo de campo en las comunidades.

### La pandemia por covid-19 en Catamarca: una breve cronología

El 11 de marzo de 2020, la Organización Mundial de la Salud (OMS) declaró que el brote del virus SARS-CoV-2 como pandemia, luego de que el número de personas infectadas por covid-19 a nivel global llegara a 118.554, y el número de muertes a 4.281, afectando hasta ese momento a 110 países. Al día siguiente, en Argentina, mediante el Decreto de Necesidad y Urgencia (DNU) No. 260/20 se estipuló la ampliación de la emergencia pública en materia sanitaria en todo el territorio nacional por el lapso de un año. Ese mismo día, el gobierno de Catamarca dispuso suspender todos los eventos y actividades masivas en la provincia y el cierre de fronteras, dejando solo habilitado el ingreso a la capital provincial, donde se montó un nodo sanitario. A los pocos días, el 17 de marzo, fueron suspendidas a nivel provincial las actividades administrativas en todo el sector público y las actividades educativas en todos los niveles. El 19 de marzo, el gobierno nacional decretaría el aislamiento social, preventivo y obligatorio (ASPO) para todo el territorio nacional.

La provincia de Catamarca fue la primera jurisdicción en el país en decretar la suspensión de las actividades administrativas del sector público y de los establecimientos educativos en todos los niveles, así como fue la primera en disponer el uso obligatorio del barbijo. Asimismo, Catamarca permaneció tempranamente con sus fronteras cerradas, ya sea el paso internacional a Chile, como las fronteras interprovinciales. Solo se permitía el ingreso de suministros esenciales como alimentos, medicamentos y combustibles, restringiendo el ingreso de personas que provinieran de zonas con circulación comunitaria del virus. En cada ingreso a la provincia se establecieron controles epidemiológicos y se fortalecieron los controles de cumplimiento de la “cuarentena” con la incorporación de personal del Ejército Argentino. 

La provincia de Catamarca no tuvo casos positivos hasta julio de 2020, momento en que a nivel nacional comenzaban a flexibilizarse algunas medidas a través de la disposición del distanciamiento social, preventivo y obligatorio (DISPO). Algunos de los elementos que pueden haber favorecido a la tardía aparición de casos son que Catamarca es una de las provincias menos pobladas (menos de 400.000 habitantes) y que no cuenta con aeropuerto internacional. A esto se podría sumar la implementación de medidas preventivas de suspensión de actividades que fueron efectuadas tempranamente.

En el caso del departamento de Santa María tuvo su primer caso positivo durante los primeros días del mes de septiembre de 2020, en un contexto de regreso parcial a cierta presencialidad en las actividades (a fines de julio de 2020 se había dispuesto el “Plan jurisdiccional de retorno a clases presenciales”). 

Cabe mencionar que un hito en esta cronología ha sido el comienzo del plan de vacunación a nivel nacional durante el mes de diciembre de 2020. Durante 2021, en el marco del avance con el plan de vacunación, en la provincia de Catamarca pueden identificarse diferentes momentos, ya sea de cierre o apertura de actividades, establecidos en función de la situación epidemiológica en los distintos departamentos (etapa “amarilla”, etapa “roja” de aislamiento estricto, etc.). A partir de julio de 2021, con un riesgo epidemiológico a nivel nacional y provincial estable o en descenso, se retornó a cierta presencialidad en la administración pública y en los diferentes establecimientos educativos.

## METODOLOGÍA

La investigación se basó en un diseño cualitativo, que prestó especial atención a las dimensiones simbólicas de la acción social y las definiciones que generan los actores sociales de los hechos relevantes para el estudio^16^^,^^17^. 

Para caracterizar la situación sociosanitaria de las comunidades seleccionadas y describir las principales medidas sanitarias y socioeconómicas implementadas por los gobiernos nacional, provincial y municipal frente a la pandemia por covid-19, se relevó información mediante fuentes primarias (entrevistas en profundidad a agentes de salud oficial) y secundarias (resoluciones y documentos oficiales; medios de comunicación; reportes sanitarios; entre otras)

Para analizar el impacto de la pandemia por covid-19 y las medidas adoptadas, se trabajó a partir de diferentes dimensiones: la sanitaria, la económica y laboral, la sociocultural, y la territorial. Respecto a la dimensión sanitaria, se buscó conocer la accesibilidad a la salud de las familias comuneras; la infraestructura y personal sanitario existente para la atención de la salud en los territorios; la existencia de políticas de salud interculturales; las experiencias respecto al covid-19 (casos positivos, tratamientos realizados, nivel de gravedad, muertes); y las posibilidades concretas de cumplimiento de las medidas sanitarias de ASPO. En relación con la dimensión económica y laboral, se indagó sobre el modo en que afectó la pandemia y las medidas específicas adoptadas en el nivel de actividad económica de las familias y comunidades diaguitas bajo estudio; su situación laboral; posibles restricciones comerciales; el nivel de ingreso familiar; la existencia de ingresos de emergencia y/o subsidios; efectos sobre trabajadores migrantes; y dificultades para la reproducción de prácticas económicas y de la reproducción de la vida vinculadas a las medidas de ASPO. Con relación a la dimensión sociocultural, se relevaron aquellos impactos en las dinámicas socioculturales de las comunidades; efectos en las prácticas socio-organizativas y en la participación de actividades culturales; impacto en los procesos educativos; efectos diferenciales según género y edad. Finalmente, respecto a la dimensión territorial, se indagó sobre el impacto de las medidas de ASPO en las situaciones de conflicto territorial y/o vinculadas a ellas, así como la participación y consulta de las comunidades en los espacios de definición de políticas sanitarias en sus territorios (por ejemplo, el Comité Operativo de Emergencia).

Paralelamente, se buscó conocer las percepciones que tienen las familias comuneras acerca del covid-19 y las medidas adoptadas, así como las diferentes estrategias construidas a nivel de las familias y comunidades diaguitas para hacer frente al covid-19 y para mitigar las consecuencias de las medidas sanitarias restrictivas implementadas. 

La entrevista en profundidad ha sido la principal herramienta metodológica utilizada, mediante la confección de una guía de pautas no estructurada que orientó las conversaciones con las personas entrevistadas. Se realizaron 15 entrevistas a personas de la comunidad, autoridades comunitarias, agentes de salud del hospital regional, agentes sanitarios, y técnicos territoriales. En forma complementaria se implementaron 30 entrevistas estructuradas a personas de la comunidad con el objetivo de explorar significados y experiencias sobre el impacto social y económico de la pandemia covid-19 en las familias y comunidades diaguitas bajo estudio. La selección de las comunidades y de las personas entrevistadas ha tenido su fundamento en criterios de factibilidad de acceso al campo y de relevancia para la investigación, ya sea por el cargo ocupado (en la comunidad o en el sistema de salud) como por el vínculo con la problemática. 

El trabajo de campo se llevó a cabo entre febrero y noviembre de 2022. Las entrevistas a agentes y funcionarios de la salud se realizaron en los centros de salud de Santa María, mientras que las entrevistas a autoridades comunitarias, agentes sanitarios indígenas y personas de la comunidad se efectuaron en las mismas comunidades, en ocasión de asambleas comunitarias y reuniones.

El análisis de los datos se llevó a cabo en forma simultánea al trabajo de campo. Por tal motivo, el proceso de análisis no ha sido considerado como una etapa diferente de la investigación, sino una actividad reflexiva presente en todo momento. Para el análisis de las entrevistas se realizó lo que Bertaux denomina “análisis comprensivo”^18^, buscando interpretar los datos obtenidos, con el fin de captar las complejidades de los mundos sociales que se propone comprender^19^. En ese marco, el sentido de lo narrado ha sido interpretado mediante la identificación de “núcleos temáticos” surgidos de categorías previamente definidas, así como de las mismas entrevistas, siguiendo un recorrido emergente. 

Por otro lado, la información relevada mediante la implementación de entrevistas estructuradas ha sido sistematizada y procesada mediante la confección de una matriz de datos, identificando características de la población bajo estudio, elementos comunes, variaciones significativas y la diversidad de perspectivas existentes sobre el objeto de investigación.

Las consideraciones éticas en el proceso de investigación han sido tomadas en cuenta desde el diseño mismo del proyecto. Se partió de vínculos construidos previamente con las comunidades y se contó con el consentimiento de sus autoridades tradicionales comunitarias para el desarrollo de la investigación desde un inicio. El proyecto de investigación (Registro Nacional de Investigaciones en Salud No. IS003480) contó con el aval del Comité de Ética del Instituto de Investigaciones Gino Germani, Argentina. Para la realización de entrevistas, se utilizó un protocolo de consentimiento informado. Con el fin de proteger la identidad de las personas entrevistadas, no se incluye información que pueda identificar a las personas entrevistadas. 

## RESULTADOS

### Impacto de la pandemia covid-19 y las medidas adoptadas en comunidades diaguitas de Santa María (Catamarca)

#### Dimensión sanitaria

El departamento de Santa María cuenta con el Hospital Zonal “Dr. Luis Alberto Vargas” (área programática No. 12) en la ciudad de Santa María y con un hospital anexo en la localidad de San José. En los parajes rurales funcionan 17 postas sanitarias dependientes del hospital de Santa María. Este hospital es de carácter regional, por lo que atiende padecimientos relativamente complejos, y frente a casos más complicados deriva hacia el hospital de la capital provincial u otras jurisdicciones de la zona con centros de salud de mayor complejidad.

Como ya se ha mencionado, las comunidades que son parte de esta investigación se encuentran mayormente en la zona serrana, manteniendo una distancia al hospital de alrededor de 80-100 km por caminos sinuosos de montaña. Trasladarse hasta los hospitales constituye una gran dificultad para las familias comuneras, no solo por las distancias que deben recorrer y los costos que eso implica, sino también por el hecho de dejar su lugar de vida y tener que adaptarse a los tiempos y posibilidades de los centros asistenciales^13^.

Según lo informado por diferentes actores en el territorio, recién en las décadas de 1980 y 1990, el sistema de salud estatal comenzó a tener presencia en los territorios de las comunidades, principalmente a partir de la creación de rutas y la mejora de los caminos^13^. En particular, mediante políticas vinculadas a la Atención Primaria de la Salud (APS), las cuales constituyen una de las pocas presencias estatales relativamente activas (junto con políticas educativas) que pueden encontrarse en los territorios diaguitas de altura. Dicha presencia se territorializa mediante la acción de agentes sanitarios y/o enfermeros del área de APS de los hospitales, quienes realizan periódicamente visitas domiciliarias o abren la posta sanitaria para llevar a cabo el control de la salud de la población, principalmente de niños, niñas y embarazadas^20^.

Durante el primer período de la pandemia, la atención sanitaria de covid-19 se concentró en el hospital de Santa María. Allí se realizaban hisopados y controles sanitarios, se atendía a personas con síntomas y se definían los aislamientos e internaciones, según el caso. En el hospital anexo de San José se atendía a polivalentes. 

Como ya se ha señalado, el covid-19 llegó de modo tardío a Santa María (el primer caso fue en septiembre de 2020), con lo cual durante una primera etapa el sistema de salud estuvo enfocado en acciones de control y prevención. Asimismo, se buscó reforzar la infraestructura y la calidad de los servicios de salud, que presentaban diferentes deficiencias al momento de inicio de la pandemia:

“…*el hospital está cambiado. Hubieses visto previo al hospital a ahora, dices: ‘este no es un hospital’. Porque modificamos infraestructura según la necesidad. Pero, también, el personal se capacitó*”. (Autoridad sanitaria, entrevista en profundidad, marzo 2022)

Con relación a la infraestructura disponible, el hospital de Santa María contó con áreas de internación diferenciadas (para polivalentes y para covid-19), y en momentos de incremento en la cantidad de casos (a mediados de 2021) se debió unificar y destinar toda la capacidad instalada a la atención de pacientes con covid-19, ya sea que fueran leves o moderados, dado que los casos de mayor gravedad eran directamente derivados a la ciudad de San Fernando del Valle de Catamarca.

“…*30 camas es lo máximo que llegamos a cubrir*. […] *en cuanto a la atención, en cuanto a la capacidad de oxígeno, nosotros podíamos atender eso: los leves que no requerían oxígeno, y los moderados que requerían poco oxígeno*”. (Autoridad sanitaria, entrevista en profundidad, marzo 2022)

Respecto a las medidas de prevención llevadas a cabo, se destaca no solo el aislamiento y el distanciamiento social preventivo y obligatorio (ASPO y DISPO), sino también el fuerte control de circulación e ingreso de personas provenientes de otras jurisdicciones, a quienes se les pedía hisopado negativo y aislamiento preventivo.

“*Pasamos mucho tiempo, a todas las personas que ingresaban, aislándolas, al principio 28 días, luego 15, luego 7 días*. […] *Los protocolos bien establecidos y causaban incomodidad en la gente que quería venir*”. (Autoridad sanitaria, entrevista en profundidad, marzo 2022)

Dichos controles de circulación afectaron fuertemente a la población que habitaba en la zona de los cerros, dado que se limitó la posibilidad de trasladarse entre los parajes y las zonas urbanizadas (movimiento que en las comunidades diaguitas es permanente, ya sea por razones laborales, de estudio, de cuidados familiares, entre otros). Asimismo, tanto los agentes de salud como las familias comuneras señalaron que, al comienzo de la pandemia, a toda persona que fuera caso positivo o sospechoso por síntomas se la aislaba en un albergue en la ciudad de Santa María, para que estuvieran cerca del centro de salud (hospital).

“*La gente estaba super en negación, porque era dejar los animales, los hijos, o a los padres. Y era desarraigarlos de golpe, pero también nosotros no teníamos como dejar de atender de acá para irnos 15 días a ver quién se deterioraba, quién sí, quién no. Entonces, lo más cercano, para nosotros, como salud, era traerlos*”. (Autoridad sanitaria, entrevista en profundidad, marzo 2022)

Esta medida fue percibida negativamente por las comunidades. Si bien varias personas entrevistadas mencionaron que en términos generales las medidas preventivas adoptadas de distanciamiento y suspensión de algunas actividades fueron importantes para que la situación de contagios no se agravara, también observaron que la decisión de aislar a los casos positivos o sospechosos lejos de su hogar, en la ciudad, no estaba considerando sus necesidades ni su realidad sociocultural al ser coaccionadas de ese modo.

“*Es como una obligación, diciendo que, si no bajábamos a Santa María que el policía iba con una nota, que el hospital se les obliga. Que se obligaban de la familia, que no querían bajar a hacer el aislamiento, digamos, en Santa María*”. (Persona de la comunidad, agente sanitaria indígena, entrevista en profundidad, agosto 2022)

A lo largo de la pandemia, desde el sistema de salud mostraron un cierto aprendizaje en el modo de abordar la situación, dado que posteriormente les ofrecieron a las personas que vivían en la zona serrana la opción de aislarse voluntariamente en su hogar mediante la firma de una declaración jurada, de modo que ya no les obligaban a dejar su comunidad.

“*Cuando fue la segunda ola, ya las personas tenían síntomas, los aislábamos, pero nada querían saber con venirse. Entonces, ellos mismos firmaban un acta voluntaria o una declaración jurada donde se querían hacer cargo, y nosotros lo que hacíamos era enviarle medicación sintomática para la fiebre, para la congestión, al agente sanitario* […] *y a los 14 días nosotros les enviábamos los certificados por WhatsApp del alta a las personas*”. (Autoridad sanitaria, entrevista en profundidad, marzo 2022)

“*A algunos los han llevado obligados, y yo conozco a mis primas… ellas han firmado un tipo acta que les ha hecho el policía, que, si pasaba algo, ellos no se responsabilizaban de ellos, del hospital, y se han quedado en su domicilio*”. (Comunera, entrevista en profundidad, agosto 2022)

Respecto de las medidas de aislamiento social, según señalan las diferentes personas entrevistadas, fueron generalmente acatadas en las diferentes comunidades bajo estudio. Asimismo, la campaña de vacunación de covid-19 se efectuó sin mayores inconvenientes, por medio de los agentes sanitarios que se instalaban en algún punto central de la comunidad o, bien, iban casa por casa a vacunar. En las comunidades hubo un alto porcentaje de personas vacunadas (con una, dos y tres dosis), según los agentes de salud entrevistados y según se pudo relevar mediante las entrevistas estructuradas que se implementaron en las diferentes comunidades. 

En relación con los contagios, algunas comunidades señalaron que prácticamente no hubo personas contagiadas o con síntomas de covid-19, mientras que otras sostenían que prácticamente todas las familias comuneras se contagiaron. Según sus relatos, se trató de casos leves, sin complicaciones. Solo en muy pocos casos requirieron traslado a la ciudad de Catamarca y al poco tiempo regresaron. Según las entrevistas y el relevamiento realizado, no hubo fallecidos entre las personas que integraban las comunidades.

Una estrategia extendida en las diferentes comunidades relevadas frente a posibles casos de covid-19 ha sido el autoaislamiento en el hogar familiar y la realización de tratamientos con “medicina tradicional”, a partir de preparados con plantas medicinales.

“*Cuando ellos tenían miedo de que nosotros los lleváramos, ellos manejaban hierbas, sus tés, y no avisaban que se sentían mal. O si no nos decían eso: ‘yo no voy a irme, yo me voy a tomar mis tés acá, denme nada más pastillas para el dolor de los huesos’, que es lo que siempre quieren, o para la fiebre*”. (Autoridad sanitaria, entrevista en profundidad, marzo 2022)

En ese sentido, ya sea para la prevención como para el tratamiento de quienes presentaban síntomas de covid-19, en las comunidades utilizaron diferentes preparados, tales como: “quemadillos” con chachacoma, raíz de espinillo, poposa, o vira vira; vino hervido con romero; caramelos de grasa de quirquincho; vahos de jarilla o eucaliptus; entre otros.

“*Gracias a Dios y la Pachamama que nosotros tenemos nuestras hierbas medicinales, así que de la puna, y con eso hemos logrado a sobrevivir*”. (Comunera, agente sanitaria indígena, entrevista en profundidad, agosto 2022)

Respecto a este tema, como se ha mencionado en trabajos previos^13^^,^^20^, las comunidades diaguitas que forman parte de la Unión de Pueblos de la Nación Diaguita de Catamarca vienen realizando un trabajo de revalorización de su medicina tradicional, luchando para que la misma sea respetada desde los centros de salud como modo propio de atención de la salud comunitaria.

[la medicina tradicional] “*no cabía en el ministerio, ni en todas las líneas de trabajo que ellos tienen. Era imposible. Pero, en realidad no es así. Falta de conocimiento de su parte. Pero, ahora, por lo menos, aflojó esa parte*”. (Agente sanitario indígena, entrevista en profundidad, octubre 2022)

Las autoridades comunitarias, agentes sanitarios y personas de la comunidad mencionan que si bien la descalificación hacia la medicina tradicional y el desconocimiento por parte del sistema de salud oficial sobre los procesos de salud-atención-cuidado en las comunidades aún persiste -lo cual genera tensiones y conflictos permanentes-, en los últimos años, mediante la lucha (inter)comunitaria, han logrado que se respeten y se reconozcan algunas prácticas y saberes propios, como es el caso del uso de algunos preparados con base en hierbas medicinales para el cuidado de la salud. 

#### Dimensión económica y laboral

Las economías de las comunidades diaguitas del departamento de Santa María están principalmente organizadas en torno a la producción agropecuaria. Las familias comuneras cuentan con una importante diversidad de producciones, todas de pequeña escala y enfocadas principalmente a garantizar el autoconsumo, aunque también llevan a cabo intercambio de sus excedentes de forma monetaria y no monetaria. Realizan principalmente cultivos andinos, gran variedad de maíces, papa, habas, quinoa, y también forrajeras, como alfalfa. También cultivan árboles frutales para consumo fresco y producción de dulces y conservas. En cuanto a la producción de ganadería, se dedican a la cría de llamas, vacas, ovejas, cabras y en menor cantidad mulas y caballos para trabajo en las comunidades. Asimismo, cuentan con aves de corral, en su mayor parte pollos y gallinas para la producción de huevos y carne. Muchas familias complementan su producción agrícola y ganadera con la elaboración de artesanías textiles en fibra de llamas, vicuña y ovejas, así como también en cueros y madera.

Si bien la actividad agropecuaria es la principal fuente de ingresos de las familias comuneras, también hay quienes se dedican a otras actividades o venden su mano de obra fuera de la comunidad, en trabajos estacionales. Aquí es importante mencionar el trabajo en la cosecha de diferentes producciones estacionales (como es el caso de la vid, el olivo, cítricos, etc.) o el trabajo de albañilería en obras de construcción.

El empleo público municipal (sin contar el empleo público provincial, ni nacional) en el departamento Santa María representaba, en 2021, cerca del 10% de la población (en Santa María había 1.134 empleados municipales y en San José 252) según datos de la Dirección Provincial de Estadísticas y Censos. Sin embargo, entre la población que conforma las comunidades bajo estudio, prácticamente no había empleadas y empleados públicos, ni tampoco era significativo el número de personas beneficiarias de programas sociales. 

A partir del relevamiento y las entrevistas realizadas, encontramos que para las comunidades diaguitas que conforman la Unión de Pueblos de la Nación Diaguita de Catamarca la pandemia por covid-19 ha tenido fuertes impactos en términos económicos y laborales, principalmente por las restricciones de circulación que tuvieron que enfrentar. Las familias entrevistadas manifiestan que buena parte de la vida productiva se mantuvo igual, por vivir con cierto aislamiento unos de otros, por trabajar en el campo con la cría de animales y los cultivos, sin necesidad de tener contactos estrechos con otras personas, e incluso, en algunos casos han ampliado su capacidad productiva. Sin embargo, encontraron serias dificultades a la hora de comercializar su producción o abastecerse de otros productos. El no poder trasladar sus producciones para la venta ha tenido un efecto significativo en las comunidades, mermando fuertemente los ingresos familiares.

“*La vida ha seguido normal en las comunidades nuestras. La máxima dificultad estuvo vinculada a las restricciones de circulación*”. (Delegado de base de comunidad, entrevista en profundidad, septiembre 2022)

Asimismo, el traslado periódico a la ciudad de Santa María, que era habitual en la vida de las familias comuneras -por razones de estudio, de trabajo o de gestiones administrativas- se vio interrumpido, afectando el circuito económico de las familias. Cabe destacar, al respecto, el efecto negativo que tuvo también para quienes trabajaban fuera de la comunidad de manera temporal en la cosecha de diferentes producciones (vid, olivo, cítricos, etc.) que se habían visto impedidos de migrar, así como para quienes se dedicaban a actividades por cuenta propia, como albañilería, comercio, etc., aunque estos casos eran minoritarios.

“*Hay jóvenes también que son trabajadores golondrinas. No han podido ir a trabajar, por ejemplo, cosechas, eso, no podían viajar a otra provincia, así que… ahí se ha visto*”. (Persona de la comunidad, entrevista en profundidad, noviembre 2022)

Como medidas compensatorias, las familias comuneras, en su mayor parte, mencionaron en las entrevistas que habían recibido poca ayuda por parte del Estado.

“*No, nada. Una sola vez cuando estábamos aislados, nos han dado un bolsón de mercadería, una bolsita de mercadería, fruta y algunas verduritas. Nada más*”.(Persona de la comunidad, agente sanitaria indígena, entrevista en profundidad, agosto 2022).

Esto es señalado como un elemento que hubiera contribuido a mitigar los efectos negativos de la imposibilidad de comercializar su producción para generar ingresos familiares. En los casos que han recibido ha sido mediante la entrega de mercadería y, en pocos casos, algún tipo de subsidio del gobierno provincial. 

#### Dimensión sociocultural

Con relación a las dinámicas socioculturales de las comunidades, a partir del relevamiento realizado encontramos que se vieron fuertemente afectadas por la suspensión de actividades culturales, religiosas, deportivas, etc. El impacto más profundo se relacionaba con los procesos educativos, que se vieron prácticamente interrumpidos en las comunidades. Las familias entrevistadas señalaron que, si bien se propusieron clases virtuales y materiales de apoyo, habían encontrado grandes dificultades dado que la mayoría de las comunidades no contaban con conectividad a Internet, ni con energía eléctrica durante todo el día (dependían de la energía solar). Las y los docentes escolares no pertenecían ni residían en las comunidades, sino que eran de la ciudad de Santa María; por lo cual, al no poder trasladarse, el acompañamiento a los procesos educativos se había vuelto aún más complejo.

“*No se hizo nada. Se suponía que les mandaban materiales, pero muy a cuentagotas, y como no teníamos Internet ni otra comunicación, ahí quedó todo*”. (Delegado de base de la comunidad, entrevista en profundidad, octubre 2022)

La mayoría de las comunidades bajo estudio tenían acceso a Internet a través de la señal de wifi de la escuela, mientras otras comunidades directamente no contaban con señal de celular ni de Internet. Solo recientemente, en algunas comunidades lograron contar con señal de Internet particular para poder conectarse en sus casas. A esta carencia, se sumó la dificultad de comprender ciertos lenguajes y herramientas tecnológicas que no resultaban apropiados para las comunidades.

“*Nosotros no tenemos luz 24 horas, y bueno, si no tenemos luz, no tenemos wifi. Y muchas familias que no tenían celular, no entienden bajar un PDF, no entienden la consigna que le dan*”. (Persona de la comunidad, entrevista en profundidad, agosto 2022)

Ya se ha mencionado que mediante las disposiciones de aislamiento y distanciamiento las comunidades se vieron obligadas a suspender todas sus actividades sociales, ya sea deportivas, religiosas, educativas, etc. Incluso, debieron suspenderse las asambleas comunitarias e intercomunitarias, afectando fuertemente la organización de la vida colectiva. De todos modos, y a pesar ello, la solidaridad, la cooperación y la reciprocidad continuó siendo un elemento fundamental para la reproducción de la vida familiar y comunitaria.

“*Por ejemplo, quedó mucha gente aislada, y yo iba a verlos. Si necesitaban algo, los llevaba, le dejaba a distancia*”. (Autoridad comunitaria, entrevista en profundidad, octubre 2022)

#### Dimensión territorial

En relación con la dimensión territorial, la articulación y la dinámica intercomunitaria diaguita se vio claramente afectada por la suspensión de actividades sociales y la restricción de circulación. Se interrumpieron las asambleas comunitarias e intercomunitarias (que se realizan todos los meses), lo cual tuvo efectos en los procesos organizativos territoriales. Más aún, en un contexto en el que ciertas actividades, como la megaminería -que representaba una amenaza para la territorialidad diaguita catamarqueña- fueron consideradas esenciales, permitiendo su continuidad. 

En las diferentes comunidades se llevaron a cabo diversos mecanismos de control territorial para evitar la circulación de personas y el ingreso y propagación del virus en sus territorios, a lo que se sumaron autoaislamientos domiciliarios en caso de personas que regresaban de la ciudad, trabajos de concientización, asistencia solidaria para las personas aisladas, etc., poniendo en juego el valor de la dimensión comunitaria.

“*El aislamiento no hacía falta porque vivimos a distancia unos de otros. Sí se recomendaba no salir hasta que estén bien, y tengan los cuidados sanitarios que establecían ellos* [...] *de todos modos, se lo advertía de que era conveniente también estar aislado y cuidarnos del otro, porque si se propagaba rápidamente era complicado*”. (Delegado de base de la comunidad, entrevista en profundidad, octubre 2022)

“*Como nosotros somos conocidos y cuando llega una camioneta nosotros la distinguimos desde lejos. Así que ahí mejor no atender*. [...] *También la cacica puso de su parte, y el agente sanitario, y el policía también. Así que ya han informado de que no podían ingresar ni vendiendo nada a la comunidad*”. (Persona de la comunidad, entrevista en profundidad, agosto 2022)

La capacidad de control territorial por parte de las comunidades se puso de manifiesto también en la participación en espacios de definición de políticas sanitarias en sus territorios, como es el caso del Comité Operativo de Emergencia (COE). En varias oportunidades, las autoridades comunitarias participaron en el COE para exigir que se respetaran o se tuvieran en cuenta las realidades de las comunidades. Por ejemplo, cuando las personas de la comunidad que habían ido a trabajar a otras provincias se quedaron sin trabajo allí y decidieron regresar a sus territorios, no les permitían el ingreso a la provincia; o para solicitar que los hospitales respetaran las decisiones de las personas de la comunidad de autoaislamiento en sus territorios; o bien, para pedir se tuvieran en cuenta los usos y costumbres y las dinámicas productivas de traslado de la hacienda de una vera a la otra del río, entre otras.

Otro ejemplo de situaciones suscitadas durante la pandemia, que da cuenta de los procesos organizativos de defensa territorial y su vínculo con la etnicidad y la salud, está vinculado al rol de los agentes sanitarios indígenas. En diferentes comunidades diaguitas de Santa María había agentes sanitarios elegidos por las mismas comunidades en el marco del Programa de Salud para Pueblos Originarios (PSPO) del Ministerio de Salud de la Nación. Este programa fue creado en 2016, a través de la Resolución 1036-E/2016, como continuación del trabajo realizado en el marco del Programa Médicos Comunitarios. Los agentes sanitarios indígenas -designados por las comunidades y avalados por las autoridades comunitarias- recibían, mediante dicho programa, un estipendio en forma de beca. Desde la Unión de Pueblos de la Nación Diaguita de Catamarca vienen luchando para que el trabajo de estos agentes sanitarios responda a las necesidades de las comunidades, mediante la práctica y fortalecimiento de la “medicina tradicional”.

Durante la pandemia, en momentos de incremento en la cantidad de casos, los directivos del hospital asignaron a algunos de estos agentes a la realización de controles en diferentes puntos de la ciudad de Santa María, para lo cual debían dejar de cumplir funciones en sus comunidades (además de alejarse de su hogar).

“*Y en la pandemia quisieron quitar el agente sanitario, en mitad de pandemia, pero era fundamental en ese tiempo*. [...] *Entonces, nosotros en ese momento no lo permitimos*”. (Autoridad comunitaria, entrevista en profundidad, septiembre 2022)

Esto generó un conflicto entre las comunidades y el sistema de salud. Las comunidades expresaron su disconformidad y debieron realizar gestiones y reclamos (a pesar de las dificultades propias del contexto) para que estos agentes sean asignados nuevamente a sus territorios. 

## DISCUSIÓN

Hemos podido identificar diversos impactos en términos sanitarios, económicos, socioculturales y territoriales producto de la pandemia por covid-19 en las diferentes comunidades diaguitas bajo estudio. Lo observado en el caso de las comunidades diaguitas en Santa María (Catamarca) coincide en buena medida con el informe realizado por Alijanati *et al.*^4^, sobre los efectos socioeconómicos y culturales de la pandemia por covid-19 y del aislamiento social, preventivo y obligatorio (ASPO) en los pueblos indígenas en Argentina realizado en 2020, en el cual se advierte una profundización en problemáticas vinculadas a la accesibilidad a la salud como ser: carencias en la infraestructura sanitaria; trato discriminatorio y racista en algunos ámbitos; escasez y/o precariedad de políticas de salud intercultural; falta de insumos médicos, de unidades de traslado y de especialistas de forma permanente; situaciones que se profundizan en contextos rurales. Es decir, la situación de pandemia ha profundizado aquellos problemas asociados a la escasa cobertura de los servicios de salud en los territorios tradicionales indígenas^1^, por el hecho de que las medidas preventivas han sido aplicadas sin un enfoque intercultural y local, trayendo aparejadas complicaciones para la vida cotidiana, la organización política y el desarrollo de la cultura de las comunidades indígenas, que han dejado de manifiesto problemas estructurales previamente existentes^1^. En este punto, varios son los estudios que enfatizan la importancia de que se incluya la dimensión intercultural y local para la implementación de medidas eficaces para hacer frente a pandemias como la del covid-19^21^.

En ese sentido, podemos vincular la escasez y precariedad de políticas de salud intercultural, la reproducción de lógicas de invisibilización y colonialidad hacia los saberes y prácticas de los pueblos indígenas con los debates en torno a las desigualdades étnicas, la discriminación y el racismo en la atención de la salud, definidos como la forma cultural-ideológica que justifica el acceso inequitativo de los pueblos indígenas a dichos recursos, a partir de factores como la inexistencia de programas específicos que atiendan sus problemáticas particulares, la dificultad para el acceso a la salud por su distribución y/o la discriminación del personal de salud a las personas usuarias por su condición étnica o cosmovisión^22^.

En un informe del Ministerio de Salud de la Nación, realizado en el marco de la consulta a los pueblos indígenas para la implementación de la “Cobertura Universal Efectiva de Salud”, se señala que:

“…en lo que respecta a los problemas relacionados con la atención de la salud, las comunidades destacan: problemas de accesibilidad tanto geográfica como económica a los servicios de salud, falta de infraestructura en postas sanitarias, escasez de personal médico (particularmente en lo que respecta a especialidades) y amplitud de días y horarios de atención, necesidad de mayor cantidad de agentes sanitarios, necesidad de un enfoque intercultural en la atención y en los establecimientos sanitarios (parto respetando la cosmología indígena, señalética bilingüe, entre otros)”.^23^

Esto mismo se ha constatado en investigaciones previas que hemos realizado con las comunidades bajo estudio, encontrando que enfrentan profundas dificultades para el acceso a la salud pública, las cuales radican en razones de diverso tipo: geográficas, debido a que se encuentran asentadas a varios kilómetros de distancia de los centros asistenciales, por caminos de montaña de difícil acceso; económicas, por el costo que implica trasladarse hasta los centros de salud en las ciudades o centros urbanos más cercanos; administrativas, dado que los turnos en los hospitales deben gestionarse en horarios específicos y de modos que son ciertamente dificultosos para la población que vive en los cerros; estructurales del sistema de salud, por la falta de infraestructuras y de personal de salud en las comunidades; culturales o simbólicas, siendo el desconocimiento y la desvalorización cultural hacia sus prácticas de curación y cuidado tradicionales uno de los principales problemas identificados^13^^,^^20^. Asimismo, si bien en los últimos años se observa un avance en la creación de políticas públicas de reconocimiento de derechos indígenas y de respeto a sus propias culturas y cosmovisiones, al mismo tiempo se evidencia la reproducción de lógicas de invisibilización y colonialidad hacia los saberes y prácticas de los pueblos indígenas en general, y la carencia de perspectivas interculturales en la atención de la salud en particular^20^.

Por otro lado, la situación sanitaria puso de manifiesto dificultades vinculadas al acceso a la educación^24^, al agua potable y la seguridad alimentaria; a la información y la justicia; a la estabilidad laboral; entre otros^1^. Es decir, en muchos casos reforzó injusticias preexistentes que afectaban los modos de producción y reproducción de la vida de las comunidades indígenas, conmoviendo todas las dimensiones de su existencia social^4^. Paralelamente, como muestra de su gran capacidad de resiliencia, las comunidades indígenas han articulado múltiples respuestas colectivas para afrontar la pandemia, entre las cuales se destacan el cierre de las fronteras territoriales comunitarias, la reciprocidad y cooperación intercomunitaria y la medicina tradicional^2^^,^^4^.

Sin embargo, vale la pena detenerse en algunos elementos particulares del caso estudiado. En términos sanitarios, identificamos que en las comunidades ha habido pocos casos de gravedad y ningún fallecido por coronavirus. Un elemento que resulta significativo es el cumplimiento al interior de las comunidades de las diferentes disposiciones preventivas (ASPO, DISPO) y el cuidado solidario para evitar la propagación del covid-19 en sus territorios. Cabe remarcar que la reproducción de la vida comunitaria diaguita está atravesada por el compartir y por la convivencialidad. Y, en este contexto de emergencia sanitaria, se priorizó la salud colectiva. El desconocimiento respecto al comportamiento de este virus y la información que circulaba generó miedo en las comunidades, que se articuló con un sentido de corresponsabilidad, pensando principalmente en el cuidado de las personas mayores, que llevó a aceptar y respetar las medidas preventivas dispuestas. 

Asimismo, es relevante señalar que una parte significativa de la reproducción de la vida en las comunidades no se vio afectada por la pandemia ni por las medidas implementadas, dado que el modo de vida en el cerro se basa en el trabajo con la hacienda, los cultivos y las tareas al interior del hogar, en parajes donde la densidad demográfica es baja y el distanciamiento social es cotidiano. El impacto mayor estuvo vinculado con dificultades en la comunicación (lo cual se vio fuertemente en el caso de la educación) y la circulación entre las comunidades y la ciudad para poder realizar, entre otras cosas, compra-venta de producciones. 

Por otro lado, cabe mencionar la importancia de la existencia de prácticas y saberes vinculados a la “medicina tradicional” para la prevención y tratamiento de covid-19 en las comunidades. En el caso de las familias y comunidades diaguitas, estas reproducen prácticas en salud, basadas en la autoatención en sus dimensiones familiares y colectivas, las cuales pueden entenderse como

“…una continuidad histórica de ejercicio sanitario vehiculizado mediante conocimientos y prácticas que son propias, devenidas de la interacción histórica con el territorio, así como otros conocimientos populares, igualmente subalternos, transmitidos de manera oral y también invisibilizados por el sistema de salud oficial como lo son los conocimientos llamados ’populares’”.^12^

Esta medicina tradicional está conformada por un amplio repertorio de saberes y prácticas terapéuticas, transmitidas hasta el día de hoy de generación en generación y que son parte de su cultura como pueblo. Según se manifiesta en las diferentes entrevistas, el uso de preparados con plantas medicinales ha sido un elemento fundamental en el cuidado y prevención de las familias comuneras para hacer frente al covid-19.

Esto asume una mayor relevancia en el marco de las dificultades de acceso al sistema de salud pública, entre las cuales cabe incluir las tensiones vinculadas a la falta de visiones interculturales en las políticas de salud y a desencuentros respecto a la atención de la salud en los territorios^13^. Es interesante observar el proceso en la construcción de estrategias para la prevención y atención de casos de covid-19 en las comunidades diaguitas, que requirió aprendizajes por parte de los diferentes actores participantes. Ante el rechazo de las comunidades a dejar su territorio y su lugar de vida para aislarse preventivamente en la ciudad, el hospital regional ha debido adaptar las estrategias, incorporando la posibilidad de que las familias comuneras pudieran optar por el aislamiento en la comunidad y practicar su propia medicina. 

El hecho de que las comunidades diaguitas pudieran afirmar su voluntad frente a decisiones de las instituciones sanitarias adquiere mayor sentido en el marco de los procesos de organización (inter)comunitaria indígena que vienen desplegándose en las últimas décadas. La reorganización de las comunidades en los últimos veinte años y su articulación en la Unión de Pueblos de la Nación Diaguita de Catamarca es manifestación de los procesos de “*r-existencia*” indígena^8^ y de la lucha por el reconocimiento del pueblo diaguita frente a las persistentes relaciones de colonialidad^5^ y subalternización de la cual han sido objeto como pueblo originario. La autogestión comunitaria es señalada por diferentes estudios^25^ como un elemento que ha permitido a las comunidades hacer frente de manera más efectiva a las dificultades que presentó el escenario pandémico, del mismo modo que la construcción de resistencias situadas^26^, a las que cabe añadir en este caso la capacidad de control territorial que han desarrollado las comunidades en favor del cuidado colectivo.

## A MODO DE CIERRE

Los resultados de la investigación brindan información acerca de los impactos sanitarios, económicos, socioculturales y territoriales de la pandemia covid-19 y las medidas implementadas para la prevención, atención y mitigación en comunidades diaguitas en el departamento de Santa María (Catamarca). Cabe destacar que es escasa la información sobre las realidades indígenas en el contexto provincial y su atención por parte del sistema de salud pública. 

En ese marco, se destaca la importancia de los procesos organizativos comunitarios e intercomunitarios que han permitido reforzar acciones de prevención y atención de la salud en los territorios, con base en saberes ancestrales y medicina tradicional, así como el establecimiento de diálogos y negociaciones con las autoridades del sistema de salud local respecto a la implementación de medidas en los territorios. Esto ha implicado aprendizajes, producto de resistencias y luchas que pusieron de manifiesto las comunidades diaguitas. Esto refuerza la idea de que resulta fundamental comprender las prácticas y estrategias desplegadas en torno a la salud en forma situada, contemplando el vínculo con el territorio y los procesos de territorialización que las mismas involucran. 

En ese sentido, el estudio evidencia, una vez más, la existencia de ciertas tensiones entre comunidades indígenas (con sus cosmovisiones y sus prácticas ancestrales) y el sistema de salud oficial, así como la relevancia que adquiere que las políticas públicas de salud se permitan comprender la realidad de los territorios y propongan revalorizar las medicinas tradicionales indígenas presentes en las comunidades, abriendo espacios donde sea posible la articulación y complementación de saberes de los distintos paradigmas en tensión: el de la medicina tradicional y la biomedicina.

En tal sentido, se propone contribuir a los debates en torno a la implementación de políticas interculturales en el ámbito de la salud para que, desde y con las comunidades indígenas en Argentina, se promuevan cambios estructurales en las relaciones de colonialidad que aún hoy están presentes en las políticas públicas y en los imaginarios sociales de la población.

## References

[1] Abeledo S, Acho E, Aljanati LI, Aliata S, Aloi J, Alonso MF (2020). Informe ampliado: efectos socioeconómicos y culturales de la pandemia COVID-19 y del aislamiento social, preventivo y obligatorio en los Pueblos Indígenas en Argentina-Segunda etapa.

[2] NU CEPAL (2020). Los pueblos indígenas de América Latina - Abya Yala y la Agenda 2030 para el Desarrollo Sostenible: tensiones y desafíos desde una perspectiva territorial.

[3] FILAC (2020). Los Pueblos Indígenas ante la pandemia del COVID-19.

[4] Alijanati L, Bompadre JM, Brown A, Castelnuovo Biraben NS, Cherñavsky SC, Colla J (2020). Sentipensarnos tierra: epistemicidio y genocidio en tiempos de COVID 19.

[5] Quijano A, Lander E (2020). La colonialidad del saber: eurocentrismo y ciencias sociales: perspectivas latinoamericanas.

[6] Lander E (2020). La colonialidad del saber: eurocentrismo y ciencias sociales: perspectivas latinoamericanas.

[7] Walsh C (2008). Interculturalidad, plurinacionalidad y decolonialidad: las insurgencias político-epistémicas de refundar el Estado. Tabula Rasa.

[8] Porto-Gonçalves WC, Ceceña A (2006). Los desafíos de las emancipaciones en un contexto miltarizado.

[9] Bengoa J (2016). La emergencia indígena en América Latina.

[10] García-Guerreiro L (2024). Después del silencio, la lucha por el territorio: Procesos de reorganización y resistencia territorial de comunidades diaguitas del departamento Santa María, Catamarca (2000-2022). Mundo Agrario.

[11] Porto-Gonçalves CW (2001). Geo-grafías: Movimientos sociales, nuevas territorialidades y sustentabilidad.

[12] Cuyul-Soto A (2013). La política de salud chilena y el pueblo Mapuche: Entre el multiculturalismo y la autonomía mapuche en salud. Salud Problema.

[13] García-Guerreiro L (2021). Prácticas y saberes médicos tradicionales del pueblo diaguita catamarqueño y su relación con el sistema de salud pública. Andes.

[14] González L, Petz MI, Barbetta PN, Calvo E, Ejarque M, Prividera G (2020). Argentina: El impacto de la pandemia covid 19 en los mundos rurales. En: Territorio y libertad: Latinoamérica rural frente a la pandemia.

[15] Alcoba LN, Salatino MN, Chavez MFF, Gonzalez L, Quiroga MB (2021). Pandemia y jóvenes en territorios rurales de Argentina. Eutopía: Revista de Desarrollo Económico Territorial.

[16] Long N (2007). Sociología del desarrollo: una perspectiva centrada en el actor.

[17] Guber R (1991). El salvaje metropolitano a la vuelta de la antropología postmoderna: reconstrucción del conocimiento social en el trabajo de campo.

[18] Kornblit A (2004). Metodologías cualitativas en ciencias sociales: modelos y procedimientos de análisis.

[19] Coffey A, Atkinson P (2005). Encontrar el sentido a los datos cualitativos: estrategias complementarias de investigación.

[20] García-Guerreiro L, Wahren J (2023). Atención Primaria de la Salud e interculturalidad en comunidades diaguitas en Salta y Catamarca, Argentina. Ciencia e Interculturalidad.

[21] Bastidas G, Báez M, Bastidas D, Bastidas G, Báez M, Bastidas D (2022). Pueblos indígenas suramericanos e interculturalidad en la pandemia de COVID-19. Memorias del Instituto de Investigaciones en Ciencias de la Salud.

[22] Cortez-Gómez R, Muñoz-Martínez R, Ponce-Jiménez P (2020). Vulnerabilidad estructural de los pueblos indígenas ante el COVID-19. Boletín sobre COVID-19.

[23] Ministerio de Salud de la Nación (2020). Marco de Planificación para Pueblos Indígenas, Proyecto BIRF “Cobertura Universal Efectiva de Salud” (P163345).

[24] Hecht AC, Enriz NM, Garcia Palacios MI (2020). Reflexiones e interrogantes acerca del impacto del aislamiento por la pandemia de COVID-19 en la educación de los pueblos indígenas de Argentina (NEA). Desidades.

[25] Campos L, Chambeaux J, Espinoza C (2021). Incidencia del COVID-19 en pueblos indígenas y afrodescendientes de Chile y la autogestión comunitaria.

[26] Wilhelmi MA (2020). Covid-19 y derechos colectivos de los pueblos indígenas: Resistencias situadas frente a la pandemia. Revista Catalana de Dret Ambiental.

