# Supplementary material for: Covid-19 y comunidades diaguitas: desigualdades estructurales y estrategias (inter)comunitarias en el departamento Santa María (Catamarca, Argentina)
Source: Salud Colect. 2024 Oct 18;20:e4868. [Article in Spanish] doi: 10.18294/sc.2024.4868 (PMC11925736; doi:10.18294/sc.2024.4868)
Supplement: Translation

# Translation: Covid-19 and Diaguita communities: structural inequalities and (inter)community strategies in the Santa María department (Catamarca, Argentina)

**Authors:** Luciana García Guerreiro

**Affiliations:** 1 Doctora en Ciencias Sociales, Becaria posdoctoral CONICET, Grupo de Estudios Rurales, Grupo de Estudios de los Movimientos Sociales de América Latina (GER-GEMSAL), Instituto de Investigaciones Gino Germani (IIGG), Universidad de Buenos Aires, Argentina. lucianagarciaguerreiro@yahoo.com.ar

**Keywords:** Homelessness, Social Stigma;Healthcare System;Spain

## Abstract

The experience of homelessness is associated with strong stigmatization processes, which are often reflected in the treatment received from professionals and the healthcare system itself. This article aims to analyze the experiences of participants in a program for homeless individuals in Barcelona called Primer la Llar within the healthcare system, and how the stigma they suffer affects the care processes. This program follows the Housing First model, a social intervention that proposes providing housing without preconditions to individuals with long histories of street living, who suffer from severe mental disorders and/or addictions. Based on individual interviews with 20 participants conducted between 2016 and 2020, it is observed that in certain cases, entering the program, having housing availability, support from professionals, and the development of their own strategies had positive effects on improving their health, although they continue to perceive discriminatory attitudes in some medical settings. It is suggested that the transformation regarding stigmatization be understood broadly, affecting individuals, institutions, and society as a whole.

## INTRODUCTION

The emergence of COVID-19 and its pandemic spread has profoundly impacted all aspects of social reproduction. The implementation of preventive social isolation measures adopted by some governments - as in the case of Argentina - necessary to prevent and mitigate the spread of infections, had effects on social life, deepening the unfathomable consequences in economic, social, psychological, and environmental aspects that the pandemic has represented for the population.

Indigenous peoples, due to their situation of structural inequality-both socio-economic and in terms of health and culture-have suffered the effects of the COVID-19 pandemic and social confinement measures to a greater extent.^1^^,^^2^^,^^3^ In this regard, it is important to understand indigenous peoples not as vulnerable sectors per se, but as individual and collective subjects whose rights have been systematically violated^4^ within the framework of colonial matrices of power and knowledge.^5^^,^^6^^,^^7^ In this way, vulnerability can be understood as a result of historical processes and social conflicts, which "translate into differential access to the material and symbolic resources necessary to practice self-care and caregiving, to promote health, to prevent infections, diseases, and deaths".^4^

This research aimed to examine how the COVID-19 pandemic has impacted Diaguita communities in the department of Santa María (province of Catamarca, Argentina), but also to account for the different strategies these communities have developed to confront the phenomenon, within the framework of territorial struggles for the recognition of their rights. At this point, the indigenous communal and organizational aspects that allow these peoples to face new adverse situations, which add to the historical processes of expropriation, marginalization, and extermination they have suffered, are emphasized. In this sense, we consider that the experiences of struggle, organizational forms, and the communal dimension that contribute to the resistance and re-existence^8^ of indigenous peoples in the region are an essential element for analyzing the effects of the COVID-19 pandemic and the measures adopted. It is worth noting that, in recent decades, within the context of "indigenous resurgence",^9^ the Diaguita communities under study have deepened their struggle for land, the political reorganization of their communities, and the recognition of their ancestral rights.^10^

At this point, we consider it essential to incorporate the notion of territory, understanding it as a geographic space shaped by social, political, cultural, and economic relationships, which is constantly redefined by the actors who inhabit and make use of it, thus configuring a territorial scenario marked by conflict over the appropriation and reterritorialization of space and natural resources, fostering the formation of identities (territorialities).^11^ In this sense, the practices and strategies carried out by indigenous communities are part of processes of appropriation and territorialization of their living space, and therefore, take on central importance within the framework of the ongoing processes of ethnic-territorial defense and recognition. Thus, the relationship between health and territory becomes fundamental.

For this same reason, we understand that health is integrated into all aspects of life and, as Cuyul Soto^12^ points out, is influenced by economic, political, environmental, spiritual, cultural, and historical conditions. Many actions in health programs for indigenous peoples have focused on addressing specific needs-needs that are precisely the result of structural and socio-historical gaps-through paternalistic and/or welfare-based approaches, which are far from promoting the exercise of autonomy and the right to self-determination of indigenous peoples.^12^^,^^13^

Regarding the effects of the COVID-19 pandemic on indigenous communities, we find that while various reports have addressed the issue at the national level,^1^^,^^4^^,^^14^^,^^15^ there is no official information on COVID-19 cases among indigenous peoples in our country, highlighting the challenges of applying an ethnic approach to health information system.^2^ In the absence of such approaches and specific information in the province of Catamarca, we set out to study six Diaguita communities in the department of Santa María (Catamarca), asking not only how the COVID-19 pandemic has impacted these rural, and particularly indigenous, communities in health, social, and economic terms, but also about the different strategies these communities have developed to face this phenomenon.

Within this framework, some of the guiding questions for this work have been: How have situations of structural and historical inequality^13^^)^ influenced the impact of the COVID-19 pandemic?; What has been the impact of the preventive and mandatory social isolation (ASPO) health measures and other public policies implemented as a result of the pandemic, and how have they been received by the communities?; What health policies have been implemented in the Diaguita territories of Catamarca to address the health needs of these communities?; What perceptions and community strategies have been developed from indigenous territories in response to this phenomenon?

### Description of the Research Context

The research focused on the Diaguita communities in the department of Santa María (Catamarca), specifically the communities of Cerro Pintao (40 families), Famabalasto (30 families), La Quebrada (12 families), La Hoyada (40 families), Toro Yaco (18 families), and Alto Valle El Cajón (79 families). According to the latest National Census of Population, Households, and Housing, in 2022, of a total of 427,625 inhabitants in the province of Catamarca, 19,668 identified as indigenous or descendants of indigenous peoples, representing 4.6% of the population. In Santa María, 5,465 people identified as indigenous out of a total of 26,822 inhabitants in the department. This means that, in 2022, 20.4% of the department's population identified as indigenous. Regarding the organizational dimension, there are over twenty Diaguita communities in the province, nine of which are located in the department of Santa María. For the purposes of this research, six of these nine communities were studied. These communities are also grouped at the provincial and regional levels into a second-level indigenous organization (Union of Diaguita Nation Peoples of Catamarca). This is an element we consider significant in organizational and territorial terms.

The communities are primarily located in what is referred to as the mountainous area, meaning the hills and high regions of the Yokavil Valley and the Alto Valle del Cajón, at an altitude of between 1,600 and 4,500 meters above sea level (Figure 1). However, a large part of the communal families maintains a constant and frequent connection with the lower area, which is more urbanized and where a significant portion of the political-administrative activities, commercial establishments, educational institutions, and health centers of the department are concentrated.

**Figure 1:** Location of the Diaguita communities under study. Department of Santa María, Catamarca, Argentina, 2022.

Source: Own elaboration.

At the time of the research, according to information provided by the communities, there were 219 families registered in the six communities under study, which represents approximately 700 people in total. It is worth mentioning that the research is framed within previously established relationships with the communities, whether through earlier research projects^10^^,^^13^ or actions that go beyond the objectives of the research (such as providing socio-organizational technical support), which constitutes a fundamental element for fieldwork in the communities.

### The COVID-19 Pandemic in Catamarca: A Brief Chronology

On March 11, 2020, the World Health Organization (WHO) declared the outbreak of the SARS-CoV-2 virus a pandemic, after the number of people infected with COVID-19 globally reached 118,554, and the number of deaths reached 4,281, affecting 110 countries at that time. The next day, in Argentina, through Emergency Decree No. 260/20, the expansion of the public health emergency throughout the national territory was stipulated for a period of one year. On the same day, the government of Catamarca decided to suspend all mass events and activities in the province and to close the borders, allowing only entry to the provincial capital, where a health node was set up. A few days later, on March 17, administrative activities in the entire public sector and educational activities at all levels were suspended at the provincial level. On March 19, the national government declared preventive and mandatory social isolation (ASPO) for the entire national territory.

The province of Catamarca was the first jurisdiction in the country to decree the suspension of administrative activities in the public sector and educational establishments at all levels, as well as being the first to mandate the use of face masks. Additionally, Catamarca promptly kept its borders closed, including the international crossing to Chile and the interprovincial borders. Only the entry of essential supplies such as food, medicine, and fuel was permitted, restricting the entry of people coming from areas with community transmission of the virus. Epidemiological controls were established at every point of entry into the province, and compliance with the "quarantine" was reinforced with the incorporation of personnel from the Argentine Army.

The province of Catamarca did not have any positive cases until July 2020, at which point some measures were beginning to be relaxed nationally through the provision of preventive and mandatory social distancing (DISPO). Some factors that may have contributed to the late emergence of cases include that Catamarca is one of the least populated provinces (with fewer than 400,000 inhabitants) and does not have an international airport. Additionally, the early implementation of preventive measures to suspend activities could also have played a role.

In the case of the department of Santa María, the first positive case was reported in the early days of September 2020, in a context of partial return to some in-person activities (by the end of July 2020, the "Jurisdictional Plan for the Return to In-Person Classes" had been established).

It is worth mentioning that a significant milestone in this chronology was the start of the national vaccination plan in December 2020. Throughout 2021, within the framework of the progress made with the vaccination plan, different moments can be identified in the province of Catamarca, whether related to the closure or reopening of activities, established based on the epidemiological situation in the various departments (yellow stage, red stage of strict isolation, etc.). Starting in July 2021, with a stable or declining epidemiological risk at the national and provincial levels, a certain degree of in-person presence returned in public administration and various educational institutions.

## METHODOLOGY

The research was based on a qualitative design that paid special attention to the symbolic dimensions of social action and the definitions generated by social actors regarding relevant events for the study.^16^^,^^17^

To characterize the socio-sanitary situation of the selected communities and describe the main health and socioeconomic measures implemented by the national, provincial, and municipal governments in response to the COVID-19 pandemic, information was gathered through primary sources (in-depth interviews with official health agents) and secondary sources (resolutions and official documents; media; health reports; among others).

To analyze the impact of the COVID-19 pandemic and the measures adopted, the research focused on different dimensions: health, economic and labor, sociocultural, and territorial. Regarding the health dimension, the aim was to understand the accessibility of health services for communal families; the existing infrastructure and healthcare personnel for health care in the territories; the existence of intercultural health policies; experiences related to COVID-19 (positive cases, treatments administered, severity levels, deaths); and the concrete possibilities for compliance with the health measures of ASPO. In relation to the economic and labor dimension, the research investigated how the pandemic and the specific measures adopted affected the economic activity level of the families and Diaguita communities under study; their employment situation; possible commercial restrictions; family income levels; the existence of emergency income and/or subsidies; effects on migrant workers; and difficulties in sustaining economic practices and life reproduction linked to ASPO measures. Concerning the sociocultural dimension, the impacts on the sociocultural dynamics of the communities were assessed; effects on socio-organizational practices and participation in cultural activities; impact on educational processes; and differential effects by gender and age. Finally, regarding the territorial dimension, the research explored the impact of ASPO measures on territorial conflict situations and/or those related to them, as well as the participation and consultation of the communities in the spaces for defining health policies in their territories (for example, the Emergency Operations Committee).

Simultaneously, the research aimed to understand the perceptions of communal families regarding COVID-19 and the measures adopted, as well as the different strategies developed at the family and community levels among the Diaguita to cope with COVID-19 and mitigate the consequences of the restrictive health measures implemented.

In-depth interviews were the main methodological tool used, through the preparation of an unstructured guideline that guided the conversations with the interviewees. A total of 15 interviews were conducted with community members, community leaders, health agents from the regional hospital, health workers, and territorial technicians. Additionally, 30 structured interviews were implemented with community members to explore meanings and experiences regarding the social and economic impact of the COVID-19 pandemic on the Diaguita families and communities under study. The selection of the communities and the interviewees was based on criteria of feasibility of access to the field and relevance to the research, whether due to the positions held (in the community or in the health system) or their connection to the issue at hand.

Fieldwork was conducted between February and November 2022. Interviews with health agents and officials were carried out in the health centers of Santa María, while interviews with community authorities, indigenous health agents, and community members were conducted in the communities themselves, during community assemblies and meetings.

The analysis of the data was conducted simultaneously with the fieldwork. For this reason, the analysis process was not regarded as a separate stage of the research but rather as a reflective activity present at all times. For the analysis of the interviews, what Bertaux refers to as "comprehensive analysis"^18^ was applied, seeking to interpret the obtained data in order to capture the complexities of the social worlds intended to be understood.^19^ In this framework, the meaning of the narratives was interpreted through the identification of "thematic cores" that emerged from previously defined categories as well as from the interviews themselves, following an emergent path.

On the other hand, the information gathered through the implementation of structured interviews was systematized and processed by creating a data matrix, identifying characteristics of the population under study, common elements, significant variations, and the diversity of perspectives regarding the research object.

Ethical considerations in the research process were taken into account from the very design of the project. The research was based on previously established relationships with the communities, and consent was obtained from their traditional community authorities from the outset. The research project (National Registry of Health Research No. IS003480) was approved by the Ethics Committee of the Gino Germani Research Institute, Argentina. For conducting interviews, an informed consent protocol was used. To protect the identity of the interviewees, no information that could identify them is included.

## RESULTS

### Impact of the COVID-19 Pandemic and the Measures Adopted on Diaguita Communities in Santa María (Catamarca)

#### Health Dimension

The Santa María department has the “Dr. Luis Alberto Vargas” Regional Hospital (Programmatic Area No. 12) in the city of Santa María and an annex hospital in the town of San José. In rural areas, there are 17 health posts dependent on the Santa María hospital. This hospital is regional in nature, so it handles relatively complex conditions, and in the case of more complicated cases, it refers patients to the provincial capital hospital or other jurisdictions in the area with higher-complexity health centers.

As already mentioned, the communities that are part of this research are mainly located in the mountainous area, maintaining a distance of around 80-100 km to the hospital along winding mountain roads. Traveling to the hospitals poses a significant challenge for community families, not only because of the distances they must travel and the associated costs but also due to the necessity of leaving their place of residence and adapting to the schedules and capabilities of the healthcare centers .^13^

According to reports from various actors in the territory, it was not until the 1980s and 1990s that the state health system began to have a presence in the communities' territories, mainly due to the creation of roads and the improvement of pathways.^13^ In particular, this was through policies related to Primary Health Care (PHC), which constitute one of the few relatively active state presences (along with educational policies) found in the high-altitude Diaguita territories. This presence is territorialized through the actions of health agents and/or nurses from the PHC departments of the hospitals, who periodically conduct home visits or open health posts to monitor the health of the population, primarily focusing on children, pregnant women, and infants.^20^

During the first period of the pandemic, COVID-19 healthcare was concentrated in the Santa María hospital. There, swabs and health checks were conducted, individuals with symptoms were attended to, and isolations and hospitalizations were determined based on each case. In the annex hospital in San José, general medical care was provided.

As previously noted, COVID-19 arrived late in Santa María (the first case was in September 2020), which meant that during the initial phase, the healthcare system focused on control and prevention actions. Additionally, efforts were made to strengthen the infrastructure and quality of health services, which had various deficiencies at the start of the pandemic:

*“…the hospital has changed. If you had seen it before and now, you’d say: ‘this is not a hospital’. Because we modified the infrastructure according to the need. But also, the staff was trained.”* (Health authority, in-depth interview, March 2022)

Regarding the available infrastructure, the Santa María hospital had separate hospitalization areas (for general cases and for COVID-19), and during periods of increased cases (in mid-2021), it had to unify and allocate all installed capacity to the care of patients with COVID-19, whether they were mild or moderate, since more severe cases were directly referred to the city of San Fernando del Valle de Catamarca.

*“…30 beds is the maximum we reached. […] in terms of care, in terms of oxygen capacity, we could manage that: the mild cases that didn’t require oxygen, and the moderate cases that required a little oxygen.”* (Health authority, in-depth interview, March 2022)

Regarding the preventive measures implemented, not only the isolation and preventive and mandatory social distancing (ASPO and DISPO) stand out, but also the strict control of circulation and the entry of people coming from other jurisdictions, who were required to present a negative test and undergo preventive isolation.

*“We spent a lot of time isolating all the people who entered, initially for 28 days, then 15, then 7 days. […] The well-established protocols caused discomfort for people who wanted to come.”* (Health authority, in-depth interview, March 2022)

The controls on circulation strongly affected the population living in the mountainous areas, as the ability to move between rural communities and urbanized areas was restricted. This movement is constant within the diaguita communities for various reasons, including work, education, and family care. Additionally, both health agents and community families noted that, at the beginning of the pandemic, any person who was a positive case or suspected of having symptoms was isolated in a shelter in the city of Santa María to be close to the health center (hospital).

*“The people were in deep denial because it meant leaving their animals, children, or parents. It was about uprooting them suddenly, but we also couldn’t just stop attending to care here for 15 days to see who deteriorated, who did, who didn’t. So, the closest thing for us, in terms of health, was to bring them.”* (Health authority, in-depth interview, March 2022)

This measure was perceived negatively by the communities. Although several interviewees mentioned that, in general terms, the preventive measures of distancing and the suspension of some activities were important in preventing the situation from worsening, they also observed that the decision to isolate positive or suspected cases away from their homes, in the city, did not take into account their needs or their sociocultural reality, as they were being coerced in that manner.

*“It’s like an obligation, saying that if we didn’t go down to Santa María, the police would come with a note, that the hospital was forcing them. They were compelled by their family, who didn’t want to go down to do the isolation, let’s say, in Santa María.”* (Community member, indigenous health agent, in-depth interview, August 2022)

Throughout the pandemic, the health system demonstrated a certain learning process in how to address the situation, as they later offered people living in the mountainous area the option to voluntarily isolate themselves at home by signing a sworn statement, so they were no longer forced to leave their community.

*“When the second wave came, people already had symptoms, and we isolated them, but they wanted nothing to do with coming down. So, they would sign themselves a voluntary act or a sworn statement taking responsibility, and what we did was send them symptomatic medication for fever and congestion through the health agent [...] and after 14 days, we would send the discharge certificates via WhatsApp to the individuals.”* (Health authority, in-depth interview, March 2022)

*"Some have been taken against their will, and I know my cousins... they signed a kind of document made by the police, stating that if something happened, they wouldn't be held responsible for them or the hospital, and they stayed at home."* (Community member, in-depth interview, August 2022)

Regarding the measures for social isolation, various interviewees indicated that they were generally followed in the different communities under study. Additionally, the COVID-19 vaccination campaign was carried out without major issues, with health agents setting up in a central location within the community or going door to door to administer vaccines. There was a high percentage of vaccinated individuals in the communities (with one, two, or three doses), according to the health agents interviewed and as recorded through the structured interviews conducted in the various communities.

In relation to infections, some communities indicated that there were practically no individuals infected or showing symptoms of COVID-19, while others maintained that nearly all communal families contracted the virus. According to their accounts, these were mild cases, without complications. Only in very few instances did individuals require transfer to the city of Catamarca, and they returned shortly thereafter. According to the interviews and data collected, there were no fatalities among the members of the communities.

An extended strategy in the different surveyed communities in response to possible COVID-19 cases has been self-isolation at home and the use of treatments involving 'traditional medicine', based on preparations made from medicinal plants.

*"When they were afraid that we would take them away, they relied on herbs, their teas, and did not inform us that they were feeling unwell. Or they would tell us: 'I’m not going to leave; I’m going to take my teas here, just give me some pills for my bone pain,' which is what they always want, or for fever."* (Health authority, in-depth interview, March 2022)

In this regard, both for the prevention and for the treatment of those presenting symptoms of COVID-19, the communities used different preparations, such as: “*quemadillos*” with *chachacoma*, *espinillo* root, *poposa*, or *vira vira*; boiled wine with rosemary; fat candies from *quirquincho*; steam inhalations of *jarilla* or eucalyptus; among others.

*“Thanks to God and Pachamama, we have our medicinal herbs from the highlands, and with that, we have managed to survive.”* (Community member, indigenous health agent, in-depth interview, August 2022)

Regarding this issue, as mentioned in previous works,^13^^,^^20^ the diaguita communities that are part of the Union of Peoples of the Diaguita Nation of Catamarca have been working on the revaluation of their traditional medicine, fighting for it to be respected by health centers as their own method of community health care.

[Traditional medicine] *“didn't fit in the ministry or in any of the lines of work they have. It was impossible. But that's not the case. It's a lack of knowledge on their part. However, at least now, that part has loosened up.”* (Indigenous health agent, in-depth interview, October 2022)

Community authorities, health agents, and community members mention that although the devaluation of traditional medicine and the lack of knowledge by the official health system regarding the health-care processes in the communities still persist -leading to ongoing tensions and conflicts- in recent years, through (inter)community struggles, they have managed to have some practices and knowledge recognized and respected, such as the use of certain herbal preparations for health care.

#### Economic and Labor Dimension

The economies of the Diaguita communities in the Santa María department are primarily organized around agricultural production. The communal families have a significant diversity of productions, all of small scale and mainly focused on ensuring self-consumption, although they also exchange their surpluses in both monetary and non-monetary forms. They mainly cultivate Andean crops, a wide variety of corn, potatoes, fava beans, quinoa, as well as forage crops like alfalfa. They also grow fruit trees for fresh consumption and for the production of jams and preserves. Regarding livestock production, they raise *llamas*, cattle, sheep, goats, and to a lesser extent, mules and horses for work within the communities. They also keep poultry, mainly chickens and hens, for egg and meat production. Many families complement their agricultural and livestock production with the creation of textile crafts made from *llama*, *vicuña*, and sheep fibers, as well as leather and wood.

While agricultural activity is the primary source of income for communal families, some individuals also engage in other activities or sell their labor outside the community for seasonal work. It is important to mention the work involved in harvesting various seasonal crops (such as grapes, olives, citrus, etc.) or construction work in masonry.

Municipal public employment (excluding provincial and national public employment) in the Santa María department accounted for approximately 10% of the population in 2021 (there were 1,134 municipal employees in Santa María and 252 in San José), according to data from the Provincial Directorate of Statistics and Censuses. However, among the population that comprises the communities under study, there were virtually no public employees, nor was there a significant number of individuals benefiting from social programs.

Based on the surveys and interviews conducted, we found that for the Diaguita communities that make up the Union of Peoples of the Diaguita Nation of Catamarca, the COVID-19 pandemic has had a significant impact in economic and labor terms, mainly due to the circulation restrictions they faced. The interviewed families expressed that much of their productive life remained the same, as they lived with a certain degree of isolation from each other and worked in the fields with animal husbandry and crops, without needing to have close contact with others. In some cases, they even expanded their productive capacity. However, they encountered serious difficulties when it came to marketing their products or obtaining other goods. The inability to transport their produce for sale had a significant effect on the communities, severely reducing family income.

*“Life has continued normally in our communities. The main difficulty was related to the circulation restrictions.”* (Community base delegate, in-depth interview, September 2022)

Additionally, the periodic travel to the city of Santa María, which was common in the lives of community families -for study, work, or administrative purposes- was interrupted, affecting the economic circuit of the families. It is important to highlight the negative impact this had on those who worked outside the community temporarily in the harvest of various products (such as grapes, olives, citrus, etc.) and were unable to migrate, as well as for those engaged in self-employed activities like construction or trade, although these cases were minor.

*“There are also young people who are seasonal workers. They haven't been able to go to work, for example, in the harvest; they couldn't travel to another province, so… that's where it has been seen.”* (Community member, in-depth interview, November 2022)

As compensatory measures, most communal families mentioned in the interviews that they had received little help from the State.

*“No, nothing. Only once when we were isolated, they gave us a bag of groceries, a small bag of groceries, fruit, and some vegetables. Nothing more.”* (Community member, indigenous health agent, in-depth interview, August 2022).

This is noted as an element that could have contributed to mitigating the negative effects of the inability to market their products to generate family income. In the cases that received assistance, it was through the delivery of goods and, in a few instances, some form of subsidy from the provincial government.

#### Sociocultural Dimension

Regarding the sociocultural dynamics of the communities, the survey conducted found that they were significantly affected by the suspension of cultural, religious, and sporting activities, among others. The most profound impact was related to educational processes, which were practically interrupted in the communities. The interviewed families noted that although virtual classes and support materials were proposed, they encountered great difficulties since most of the communities lacked internet connectivity and did not have electricity throughout the day (they depended on solar energy). The school teachers did not belong to or reside in the communities; they were from the city of Santa María. Therefore, as they could not travel, the support for educational processes became even more complex.

*“Nothing was done. It was supposed that they would send materials, but very sporadically, and since we didn’t have the internet or any other means of communication, everything just stopped.”* (Community base delegate, in-depth interview, October 2022)

Most of the communities under study had access to the Internet through the school's Wi-Fi signal, while others had no cellular or Internet signal at all. Only recently did some communities manage to have their own Internet signal to connect at home. This lack was compounded by the difficulty of understanding certain languages and technological tools that were not suitable for the communities.

*“We don’t have electricity 24 hours a day, and well, if we don’t have electricity, we don’t have Wi-Fi. Many families that didn’t have a cellphone don’t understand how to download a PDF; they don’t understand the instructions given to them.”* (Community member, in-depth interview, August 2022)

It has already been mentioned that through the isolation and distancing provisions, the communities were forced to suspend all their social activities, whether sports, religious, educational, etc. Community and inter-community assemblies also had to be suspended, significantly affecting the organization of collective life. Nonetheless, despite this, solidarity, cooperation, and reciprocity continued to be fundamental elements for the reproduction of family and community life.

*“For example, many people were isolated, and I would go to see them. If they needed anything, I would bring it to them, leaving it at a distance.”* (Community authority, in-depth interview, October 2022)

#### Territorial Dimension

Regarding the territorial dimension, the articulation and intercommunity dynamics of the Diaguita were clearly affected by the suspension of social activities and the restriction of movement. Community and intercommunity assemblies (which are held monthly) were interrupted, impacting territorial organizational processes. Moreover, in a context where certain activities, such as large-scale mining -which posed a threat to Diaguita territoriality in Catamarca- were deemed essential, allowing them to continue.

In the different communities, various territorial control mechanisms were implemented to prevent the movement of people and the entry and spread of the virus in their territories. This was complemented by self-isolation at home for those returning from the city, awareness-raising efforts, and solidarity assistance for isolated individuals, thus highlighting the importance of the community dimension.

*"Isolation wasn’t necessary because we live far from each other. However, it was recommended not to go out until people were well and had followed the health guidelines they established [...] In any case, it was advised that isolation was also a good idea, and that we should protect ourselves from one another, because if it spread quickly, it would be complicated."* (Base delegate of the community, in-depth interview, October 2022)

*"Since we all know each other, when a truck arrives, we can recognize it from a distance. So, it’s better not to engage with them [...] The cacique also did their part, as did the health agent and the police. They already informed everyone that no one could enter the community, not even to sell anything."* (Community member, in-depth interview, August 2022)

The communities' capacity for territorial control was also evident in their participation in spaces where health policies for their territories were defined, such as the Emergency Operations Committee (COE). On several occasions, community authorities participated in the COE to demand that the realities of the communities be respected or considered. For example, when community members who had gone to work in other provinces lost their jobs and decided to return to their territories, they were not allowed to enter the province. They also advocated for hospitals to respect the decisions of community members to self-isolate in their territories and requested that local customs and practices, such as the productive dynamics of moving livestock from one side of the river to the other, be taken into account, among other issues.

Another example of situations that arose during the pandemic, highlighting the organizational processes of territorial defense and their connection to ethnicity and health, is related to the role of Indigenous health agents. In various Diaguita communities in Santa María, there were health agents chosen by the communities themselves under the National Ministry of Health’s Health Program for Indigenous Peoples (PSPO). This program was created in 2016 through Resolution 1036-E/2016, as a continuation of the work carried out under the Community Doctors Program. The Indigenous health agents-designated by the communities and endorsed by community authorities-received a stipend through the program in the form of a scholarship. The Union of Peoples of the Diaguita Nation of Catamarca has been advocating for the work of these health agents to address the needs of the communities, through the practice and strengthening of "traditional medicine."

During the pandemic, when there was an increase in the number of cases, hospital administrators assigned some of these agents to perform monitoring tasks at various points in the city of Santa María. This required them to stop fulfilling their duties in their communities and to leave their homes.

*"And during the pandemic, they wanted to remove the health agent, right in the middle of the pandemic, but they were essential at that time. [...] So, at that moment, we didn’t allow it."* (Community authority, in-depth interview, September 2022)

This created a conflict between the communities and the health system. The communities expressed their dissatisfaction and had to engage in management and complaints (despite the challenges posed by the context) to ensure that these agents were reassigned to their territories.

## DISCUSSION

We have been able to identify various impacts in terms of health, economic, sociocultural, and territorial factors resulting from the COVID-19 pandemic in the different Diaguita communities under study. What has been observed in the case of the Diaguita communities in Santa María (Catamarca) largely aligns with the report by Alijanati et al.^4^ on the socioeconomic and cultural effects of the COVID-19 pandemic and the mandatory social preventive isolation (ASPO) on Indigenous peoples in Argentina, conducted in 2020. This report highlights a deepening of issues related to health accessibility, such as: deficiencies in healthcare infrastructure; discriminatory and racist treatment in some areas; scarcity and/or precariousness of intercultural health policies; lack of medical supplies, transportation units, and permanent specialists; situations that are exacerbated in rural contexts. In other words, the pandemic situation has intensified problems associated with the limited coverage of health services in traditional Indigenous territories,^1^ due to the fact that preventive measures have been applied without an intercultural and local approach, leading to complications for daily life, political organization, and the cultural development of Indigenous communities, which have revealed previously existing structural problems.^1^ In this regard, several studies emphasize the importance of including the intercultural and local dimension for the effective implementation of measures to address pandemics like COVID-19.^21^

In this sense, we can link the scarcity and precariousness of intercultural health policies, the reproduction of logics of invisibility and coloniality regarding the knowledge and practices of Indigenous peoples, to debates surrounding ethnic inequalities, discrimination, and racism in healthcare. These issues are defined as the cultural-ideological form that justifies the inequitable access of Indigenous peoples to such resources, stemming from factors such as the lack of specific programs that address their particular issues, difficulties in accessing health services due to their distribution, and the discrimination of healthcare personnel against users based on their ethnic condition or worldview.^22^

In a report from the National Ministry of Health, conducted as part of the consultation with Indigenous peoples for the implementation of “Effective Universal Health Coverage,” it is noted that:

"...regarding the problems related to healthcare, the communities emphasize: geographical and economic accessibility issues to health services, lack of infrastructure in health posts, shortage of medical personnel (particularly regarding specialties), limited days and hours of service, the need for a greater number of health agents, and the necessity for an intercultural approach in healthcare and health facilities (respecting Indigenous cosmology during childbirth, bilingual signage, among others)."^23^

This has also been observed in previous research we have conducted with the communities under study, finding that they face deep difficulties accessing public health services, which stem from various reasons: geographical, as they are located several kilometers away from healthcare centers, accessible only by difficult mountain paths; economic, due to the costs associated with traveling to the nearest health centers in cities or urban areas; administrative, since appointments at hospitals must be managed during specific hours and in ways that are quite challenging for the population living in the hills; structural, due to the lack of infrastructure and health personnel in the communities; and cultural or symbolic, with the ignorance and devaluation of their traditional healing and care practices being one of the main identified problems.^13^^,^^20^ Moreover, although there has been progress in recent years in creating public policies that recognize Indigenous rights and respect their cultures and worldviews, there is simultaneously evidence of the reproduction of logics of invisibility and coloniality regarding the knowledge and practices of Indigenous peoples in general, as well as the lack of intercultural perspectives in healthcare in particular.^20^

On the other hand, the health situation highlighted difficulties related to access to education,^24^ potable water, and food security; information and justice; job stability; among others.^1^ In other words, in many cases, it reinforced pre-existing injustices that affected the production and reproduction of life in Indigenous communities, impacting all dimensions of their social existence.^4^ At the same time, as a demonstration of their great resilience, Indigenous communities have articulated multiple collective responses to confront the pandemic, among which the closure of community territorial borders, intercommunity reciprocity and cooperation, and traditional medicine stand out.^2^^,^^4^

However, it is worth pausing to consider some specific elements of the case studied. In health terms, we identified that there have been few severe cases in the communities and no deaths from coronavirus. A significant factor is the adherence within the communities to the various preventive measures (ASPO, DISPO) and the mutual care to avoid the spread of COVID-19 in their territories. It is important to highlight that the reproduction of Diaguita community life is characterized by sharing and conviviality. In this context of a health emergency, collective health was prioritized. The lack of knowledge about the behavior of this virus and the information circulating generated fear in the communities, which was intertwined with a sense of shared responsibility, primarily focused on the care of elderly individuals, leading to the acceptance and respect for the preventive measures put in place.

It is also relevant to note that a significant part of life in the communities was not affected by the pandemic or the measures implemented, as the way of life in the hills is based on work with livestock, crops, and household tasks, in areas where the population density is low and social distancing is a daily practice. The major impact was related to difficulties in communication (which was strongly felt in the case of education) and movement between the communities and the city to conduct, among other things, the buying and selling of products.

On the other hand, it is important to mention the significance of practices and knowledge related to "traditional medicine" for the prevention and treatment of COVID-19 in the communities. In the case of Diaguita families and communities, these reproduce health practices based on self-care in their family and collective dimensions, which can be understood as

“…a historical continuity of health practices conveyed through knowledge and practices that are their own, stemming from the historical interaction with the territory, as well as other popular knowledge, equally marginalized, transmitted orally and also rendered invisible by the official health system, such as the so-called 'popular' knowledge.”^12^

This traditional medicine comprises a wide repertoire of knowledge and therapeutic practices, transmitted from generation to generation to this day, and is an integral part of their culture as a people. As expressed in the various interviews, the use of preparations with medicinal plants has been a fundamental element in the care and prevention strategies of community families to confront COVID-19.

This becomes increasingly relevant in the context of difficulties accessing the public health system, which include tensions related to the lack of intercultural perspectives in health policies and misunderstandings regarding healthcare in the territories.^13^ It is interesting to observe the process of constructing strategies for the prevention and care of COVID-19 cases in Diaguita communities, which required learning from the different participating actors. In response to the communities' refusal to leave their territory and place of residence to isolate preventively in the city, the regional hospital had to adapt its strategies, incorporating the option for community families to choose isolation within the community and practice their own medicine.

The fact that the Diaguita communities could assert their will in the face of decisions made by health institutions makes greater sense within the framework of (inter)community Indigenous organization processes that have unfolded over the past few decades. The reorganization of the communities in the last twenty years and their articulation within the Union of Diaguita Peoples of Catamarca is a manifestation of Indigenous "r-existence" processes^8^ and the struggle for the recognition of the Diaguita people in the face of the persistent relationships of coloniality^5^ and subalternization that they have been subjected to as an Indigenous people. Community self-management is highlighted by various studies^25^ as an element that has allowed communities to more effectively confront the difficulties presented by the pandemic scenario, much like the construction of situated resistances,^26^ to which we can add in this case the territorial control capacity that the communities have developed in favor of collective care.

## CONCLUSION

The results of the research provide information about the health, economic, sociocultural, and territorial impacts of the COVID-19 pandemic and the measures implemented for prevention, care, and mitigation in Diaguita communities in the Santa María department (Catamarca). It is important to highlight that there is limited information about Indigenous realities in the provincial context and their attention by the public health system.

In this context, the importance of community and intercommunity organizational processes is highlighted, which have allowed for the reinforcement of health prevention and care actions in the territories, based on ancestral knowledge and traditional medicine, as well as the establishment of dialogues and negotiations with the authorities of the local health system regarding the implementation of measures in the territories. This has involved learning, resulting from the resistances and struggles demonstrated by the Diaguita communities. This reinforces the idea that it is essential to understand the practices and strategies deployed around health in a situated manner, considering the link with the territory and the processes of territorialization that they involve.

In this regard, the study highlights, once again, the existence of certain tensions between Indigenous communities (with their worldviews and ancestral practices) and the official health system, as well as the importance of public health policies understanding the reality of the territories and proposing to revalue the traditional Indigenous medicines present in the communities, creating spaces where the articulation and complementarity of knowledge from the different paradigms in tension-the traditional medicine and biomedicine-can occur.

In this sense, it is proposed to contribute to the debates surrounding the implementation of intercultural policies in the health sector so that, with and from Indigenous communities in Argentina, structural changes in the coloniality relationships that are still present in public policies and in the social imaginaries of the population are promoted.
